# 4226 Poor provider-patient communication, lack of readiness for discharge, and perceived illness threat are associated with quality of life after survival from cardiac arrest

**DOI:** 10.1017/cts.2020.419

**Published:** 2020-07-29

**Authors:** Alex Presciutti, Jonathan Shaffer, Mary Newman, Sarah Perman

**Affiliations:** 1 University of Colorado Denver

## Abstract

OBJECTIVES/GOALS: Studies have shown that cardiac arrest survivors have poor quality of life (QoL) secondary to neurologic injury. We hypothesized that poor provider-patient communication, lack of readiness for discharge, and perceived illness threat would be associated with QoL in cardiac arrest survivors. METHODS/STUDY POPULATION: We distributed an online survey to the Sudden Cardiac Arrest Foundation listserv. Survivors completed the Questionnaire for the Quality of Provider-Patient Interactions (QQPPI), Readiness for Hospital Discharge Scale (RHDS), and the Brief Illness Perception Questionnaire (B-IPQ). When completing the QQPPI and RHDS, survivors were asked to think back to their hospitalization and discharge. QoL domains (physical, psychological, social) were measured via the WHO-QOL BREF. Three multiple regression models examined associations between QQPPI, RHDS, and B-IPQ scores with QoL domains, adjusted for age, sex, months since arrest, and understanding of arrest and post-arrest symptoms at discharge. RESULTS/ANTICIPATED RESULTS: A total of 163 survivors (mean age 50.1 years, 50.3% women) provided complete survey data. Greater perceived illness threat (β: −.45, p < .001) and lower readiness for discharge (β: .22, p = .01) were associated with worse physical QoL; greater perceived illness threat (β: −.45, p < .001) was associated with worse psychological QoL; and greater perceived illness threat (β: −.3, p < .001) and poor provider-patient communication (β: .35, p < .001) were associated with worse social QoL. Our models explained 48%, 43%, and 30% of the variance in physical, psychological, and social QoL, respectively (p < .001). DISCUSSION/SIGNIFICANCE OF IMPACT: In-hospital interactions and perceived illness threat have important ramifications for cardiac arrest survivors attempting to return to daily life. Discussions regarding cardiac arrest sequelae, expectations, and specific treatment options during hospitalization could impact future QoL.

